# Infiltrating macrophages and interferon gamma rather than renal genotype dictate heightened crescentic glomerulonephritis

**DOI:** 10.3389/fimmu.2024.1484525

**Published:** 2024-12-19

**Authors:** Yong Du, Yuyang Fu, Yuyang Gao, Dugyala Poojitha, Vanarsa Kamala, Quanzhen Li, Xinjing Zhou, Chandra Mohan

**Affiliations:** ^1^ College of Medicine, The Pennsylvania State University, Hershey, PA, United States; ^2^ Department of Rheumatology, University of Texas Southwestern Medical Center, Dallas, TX, United States; ^3^ Department of Biomedical Engineering, University of Houston, Houston, TX, United States

**Keywords:** macrophage, interferon-gamma, crescentic glomerulonephritis, intrinsic renal cells, renal transplant

## Abstract

**Background:**

Both intrinsic renal cells and immune cells contribute to driving renal inflammation and damage. However, the respective roles of intrinsic renal cells and immune cells in crescentic glomerulonephritis, and the key molecular factors driving pathogenesis are still unclear.

**Methods:**

The roles of intrinsic renal cells and renal infiltrating immune cells in crescent formation were explored using renal transplantation after experimental anti-GBM disease induction in 129x1/svJ and C57BL/6J mice. Both strains share MHC, but vary in anti-GBM nephritis susceptibility. The role of macrophage and IFN-γ in crescent formation was investigated using adoptive transfer of macrophages with altered IFN-γ expression. The gene expression profile difference between 129x1/svJ and C57BL/6J macrophages was compared using Affymetrix arrays and Gene Ontology (GO) enrichment analysis.

**Results:**

B6 recipient mice transplanted with 129x1/svJ kidneys were resistant to anti-GBM challenge, as evidenced by stable renal function and less severe renal pathology. Conversely, 129x1/svJ recipient mice receiving B6 kidneys developed severe renal damage with crescent formation, comparable to the disease in parental 129x1/svJ mice. 129x1/svJ macrophages exhibited heightened IFN-γ and IFN-γ related gene expression compared to B6 macrophages. Adoptive transfer of 129x1/svJ macrophages with subdued IFN-γ expression reduced anti-GBM nephritis, while B6 macrophages with up-regulated IFN- γ expression worsened renal damage.

**Conclusion:**

Using renal transplantation between 129x1/svJ and C57BL/6J mice and anti-GBM disease induction, we found infiltrating immune cells, not intrinsic renal cells, to play the dominant role in initialing and driving glomerular crescent formation. In particular, macrophage IFN-γ expression was critical for crescent formation.

## Introduction

Crescentic glomerulonephritis (CresGN) is a severe form of kidney disease characterized by the formation of crescent-shaped lesions in renal glomeruli. Although the detailed mechanisms of how a crescent forms and which factors contribute to its development are still not fully elucidated, it is clear that proliferating glomerular epithelial cells are the main cellular components of crescents (1). Considerable evidence from both *in vivo* and *in vitro* studies has shown that both intrinsic renal cells and immune cells play critical roles in driving renal pathological changes and damage. Indeed, intrinsic renal cells play dual roles in renal pathogenesis, acting as both victims and assailants through various mechanisms, such as inflammatory cytokine production, extracellular matrix deposition, and epithelial-mesenchymal transition (2). However, which cell types, intrinsic renal cells, or immune cells, play a dominant role or initiate renal injury still remains unclear. A recent study proposed a two-step “outside-in and inside-out” integrated model to explain the pathogenesis of crescent formation, in which immune-mediated glomerular endothelial injury first leads to inside-out damage to the glomerulus, and then subsequent leukocyte migration through the weakened or ruptured Bowman’s capsule resulted in secondary outside-in injury (3). The study implicated immune cell-mediated glomerular injury as the initial trigger. However, many questions still remain.

Among the many immune cells, the role of macrophages in meditating renal damage has been extensively examined. Macrophage accumulation is a hallmark of both human GN and various animal renal disease models, and the degree of macrophage infiltration is closely associated with the severity of clinicopathologic phenotypes (4–7). In rat anti-GBM nephritis, adoptive transfer of Bone marrow-derived (BM) or NR8383 macrophages directly worsened renal injury, as evidenced by increased proteinuria and mesangial cell proliferation (8). The same group further showed that these macrophages could home into inflamed kidneys, and that macrophage activation status was more critical than macrophage numbers in causing renal injury (9). Nevertheless, reducing or eliminating macrophages using different strategies led to improved renal injury, renal function and desirable outcomes (3, 4, 7)

Anti-GBM nephritis is a stereotype autoantibody-induced GN characterized by crescent formation and rapid renal function deterioration. The disease can be reliably induced in mice using anti-GBM antibodies or “nephrotoxic serum”. Our previous works have demonstrated GN phenotype variation across more than 20 inbred stains, following exposure to anti-GBM antibodies (10, 11). One of our key findings was that the 129x1/svJ strain was susceptible to anti-GBM serum challenge, with severe crescent formation and worsened renal function. In contrast, an MHC-matched strain, C57BL/6J (B6), was relatively resistant to anti-GBM nephritis, presenting with stable renal function and improved renal damage after the same degree of challenge. Although the detailed mechanism of this observed difference is not clear, genetic differences between these two strains are assumed to be at play.

In the current study, we pursue this strain difference deeper. To understand if the heightened susceptibility of the 129x1/svJ strain to anti-GBM nephritis is encoded in its renal genotype or immune system, we first performed renal transplantation between 129x1/svJ and B6 mice, two strains with matched-MHC background, then subjected the recipients, 129x1/svJ mice with B6 kidneys, or conversely, B6 mice with 129x1/svJ kidneys, to anti-GBM nephritis. The results clearly showed that the 129x1/svJ immune system, not intrinsic renal cells, plays a dominant role in the development of GN. Using adoptive transfer of macrophages with altered IFN- γ expression, our work also demonstrates that infiltrating macrophages and IFN- γ are the key modulators in crescentic glomerulonephritis.

## Material and methods

### Mice

C57BL/6J (B6) and 129x1/svJ mice were obtained from the Jackson Laboratory (Bar Harbor, ME, USA). All mice used for this study were bred and housed in a specific pathogen-free colony at UT Southwestern Medical Center, Department of Animal Resources, in Dallas, TX, USA. All experimental procedures were approved by the IACUC at the UT Southwestern Medical Center.

### Renal transplantation

Vascularized kidney transplantation was performed using a protocol described previously (12). Briefly, the mouse was shaved and anesthetized with an intraperitoneal injection of Sodium Pentobarbital (100 mg/kg) mixed with xylazine (10 mg/kg). The donor’s suprarenal and infrarenal aorta and vena cava were exposed and dissected, then the left ureter was dissected free and cut at a level close to the bladder. The donor kidney was then perfused with heparinized normal saline. After perfusion, the renal artery was transected with a small aortic cuff, and the renal vein was transected at its junction. The donor’s aortic cuff and the renal vein were trimmed carefully in a cold plate containing the University of Wisconsin (UW) solution before transplantation. The recipient was prepared by exposure of the infrarenal aorta and vena cava and isolation of approximately 1.0 cm of these vessels. The donor’s aortic cuff and renal vein were anastomosed end-to-side to the recipient’s abdominal aorta and vena cava using 11-0 suture continuously. To minimize warm ischemia injury, the donor kidney was covered with cold sponges throughout the procedure. After reperfusion of the donor kidney, the recipient’s bladder was dissected, and the donor ureter was pulled into the recipient’s bladder and fixed on the exterior wall. The abdomen was closed using 6-0 suture, and the recipient was placed on a heating pad to maintain the body temperature at 37°C to 38°C. The right native kidney was removed at the time of grafting and the left native kidney was removed 3 days later. All recipient mice were fed with drinking water containing sulfatrim (100mg/kg) until the mice were sacrificed. 4-6 weeks after transplantation, all recipient mice were subjected to blood urea nitrogen (BUN) and 24hr proteinuria (PU) testing before initiating the anti-GBM serum challenge studies.

### Anti-GBM nephritis model

Rabbit anti-GBM serum was purchased from Lampire Biological Laboratories (Pipersville, PA, USA), as described previously (10). Rabbit IgG and complete Freud’s adjuvant (CFA) were obtained from Sigma. The procedure to induce anti-GBM nephritis has been described in detail elsewhere (10, 11). Simply, five days after immunization with rabbit IgG with CFA, mice were injected with rabbit-anti-mouse GBM serum at a dose of 150ug. In all studies, we collected serum for BUN and serum creatinine (sCr) detection. BUN was measured using a commercially available kit (Sigma Chemicals), and sCr was detected using a capillary electrophoresis diode array detector. 24-hr urine samples were collected using metabolic cages, and the total amount of urinary protein was measured using a Coomassie blue-based assay (Pierce, Rockford, IL, USA).

For all mice undergoing renal transplantation, we monitored their renal function and 24-hr proteinuria once per week for 4-6 weeks after transplantation. Only recipients with stable renal function and negative urine protein were subjected to the anti-GBM serum challenge.

### Renal pathology evaluation and macrophage staining

At the end of each experiment, kidneys were fixed, sectioned, and stained with hematoxylin and eosin (H&E) and Periodic Acid Schiff (PAS). At least 20 glomeruli were examined per section by light microscopy for evidence of inflammation and/or tissue damage and graded in a blinded fashion as described before (10–14). The occurrence of any mesangiopathic, capillary hyaline, and proliferative, membranous or crescentic glomerular changes were also noted.

Formalin-fixed paraffin-embedded sections were used for macrophage staining. Briefly, after de-paraffinization and rehydration, heat-induced antigen unmasking was performed using citrate buffer pH 6.0. Then, the section was incubated with anti-F4/80 (Sant Cruz 1:400) overnight. The secondary Ab conjugated with peroxidase was incubated with the sections at room temperature for 2 hr. The sections were finally visualized using a commercial DAB Kit.

### Macrophage and DCs culture, labeling, and adoptive transfer

Single-cell suspensions of bone marrow (BM) were cultured with M-CSF, or IL-4 plus GM-CSF (10 ng/ml for all cytokines; R&D Systems) for macrophage and DC expansion, respectively. Low-endotoxin FBS (HyClone) was used, and endotoxin contamination of culture medium was excluded using the LAL QCL-1000 kit from BioWhittaker Inc (Walkersville, MD). Seven days after culture, the composition of the cultured cells was ascertained using flow cytometry, using Abs to CD11b, CD11c, F4/80, and Gr-1. Cultured cells were then enumerated and used for various *in vitro* or *in vivo* studies, as indicated. For *in vivo* adoptive transfer studies, 1x 10^6^ cells were administrated into each mouse in 150 µl PBS by tail vein injection.

To examine phenotype difference between 129x1/svJ and B6 macrophages, R &D duoset ELISA kits were used to detect IFN-γ and IL-1β levels in macrophage culture supernatants. To track macrophages *in vivo* after i.v injection, 129 macrophages were labeled with CM-DiI (CellTrackerTM, Invitrogen, USA), a fluorescent membrane label, *in vitro* before injection, according to the manufacturer’s instruction. Briefly, 2 µM CM-DiI was added to 1x 10^6^ macrophages, followed by 5 minutes of incubation at 37°C, and then for an additional 15 minutes at 4°C. After labeling, cells were harvested into a serum-free medium immediately before injection and injected 24 hr after the anti-GBM serum challenge. Kidneys were collected at 30 min, 24 hr, 3 days, and 7 days after macrophage transfer, and frozen sections were made. Kidney tissue slides were visualized under fluorescence microscopy, and transferred macrophages appeared as red fluorescent cells.

### Knockdown of IFN-γ expression in 129x1/svJ macrophages using Morpholinos

**Figure 1:**
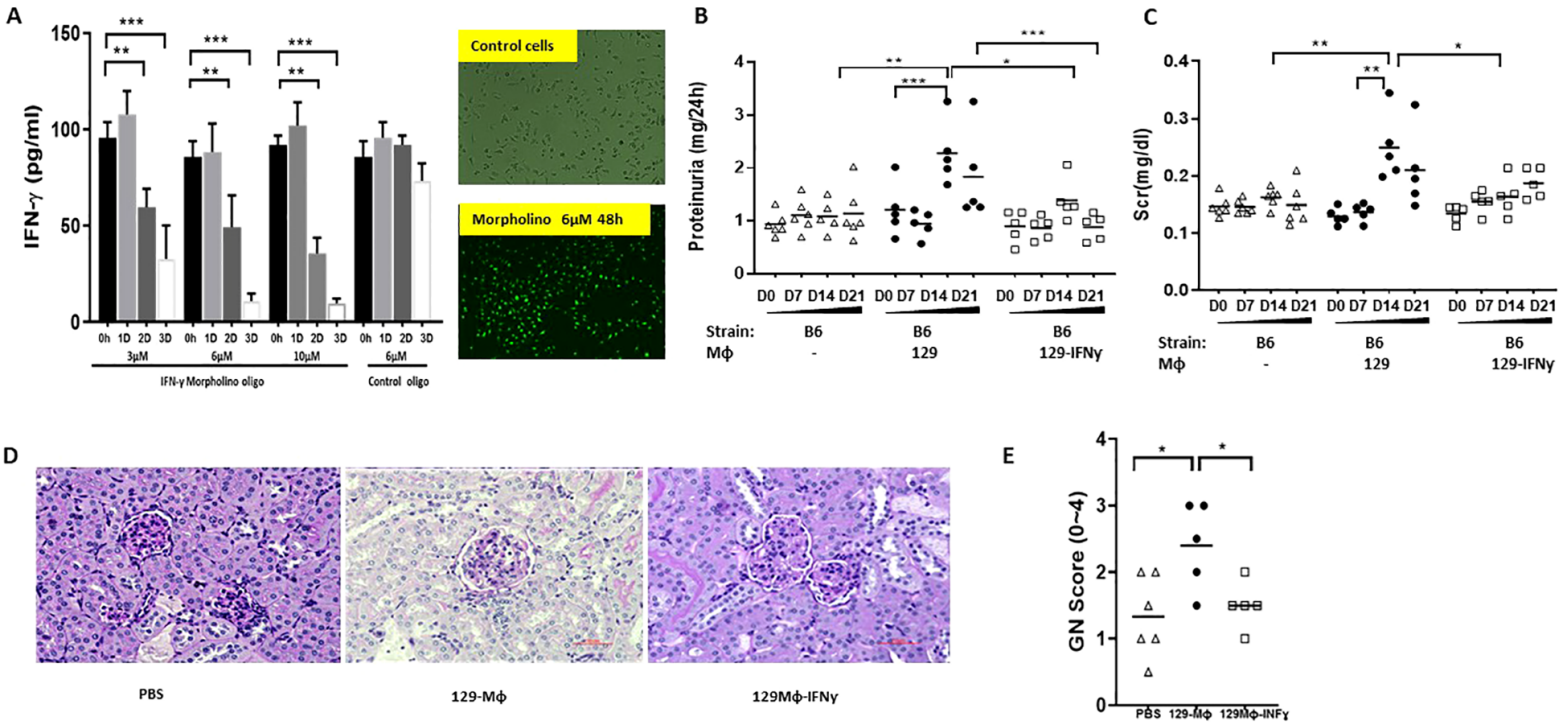
Down-regulating INF-γ expression in 129x1/svJ macrophages alleviates immune-mediated nephritis. **(A)** down-regulated IFN-γ production using anti-sense oligo; **(B)** 24hr proteinuria; **(C)** sCr; **(D)** Representative renal pathology images; **(E)** GN score. BMDMs were cultured from 129x1/svJ bone marrow with MCSF 10ng/ml for 5 days, then transfected with 6µM Morpholinos and 4µM endo-porter for 3 days. Then, these cells and non-transfected 129x1/svJ macrophages were used for *in vivo* transfer studies. Non-transfected 129x1/svJ BMDMs or IFN-γ expression down-regulated BMDMs were administrated to B6 mice subjected to anti-GBM disease 24 hr after anti-GBM serum injection. The administration of non-transfected 129x1/svJ BMDMs worsened renal function and pathology, as indicated by higher 24hr proteinuria **(B)** and sCr levels **(C)** on D14 and D21, whereas the administration of 129x1/svJ BMDMs with suppressed IFN-γ expression led to significantly decreased 24hr proteinuria and sCr on Day 14 and Day 21 (n= 5-6 per group, two-tailed t-test, * p <0.05, ** p < 0.01, *** p < 0.001).

Antisense Morpholino oligonucleotides (MO) (Gene-Tools) were used to knock down IFN-γ expression in 129x1/svJ macrophage. We hypothesize that 129 x1/svJ macrophages with lower IFN-γ would demonstrate a less inflammatory phenotype when compared to those with enhanced IFN-γ expression. The Morpholinos were designed to target IFN-γ (Gene ID NM_008337.3) translation initiation sequence, with the following sequence: 5’-CCAAGATGCAGTGTGTAGCGTTCAT-’3. A mismatched sequence Morpholino conjugated with FITC was used as the negative control, with the following sequence: 5’-CCtAcATGgAGTGTcTAcCGTTCAT-3. The efficacy of IFN-γ knockdown was assessed by both immunofluorescence microscopy and ELISA assaying the supernatant for IFN-γ levels. After the initial pilot test, we found 6µM Morpholinos with 4µM endo-porter for 3 days’ incubation yielded the maximum IFN-γ inhibition (**Figure 1A**), with the best cell visibility. Hence, 6µM Morpholinos with 4µM endo-porter were co-cultured with 1x 10^6^ cells/each plate for 3 days. Then, these cells, along with unmanipulated 129x1/svJ macrophages, were used for *in vivo* transfer studies.

### Enhanced IFN-γ expression in B6 macrophages using adenovirus

IFN-γ expression in B6 macrophages was up-regulated using adenoviral construct. The recombined adenovirus (E1 and E3 deleted adenovirus Type 5 containing mouse interferon-γ cDNA insert), and negative control adenovirus containing GFP were ordered from Vector Biolabs (Philadelphia, PA). Human cytomegalovirus (CMV) promoter-driven IFN-γ and GFP expression system was chosen. When macrophages were confluent, the cells were inoculated with viral particles. After one hour of incubation, the medium was replaced with new medium. Three ratios, 100:1, 50:1 and 10:1 of viral particles *to* target macrophages, were tested, and 50:1 ratio was chosen as over 90% of macrophages expressed GFP. After two days of culture at 37 ˚C in a 5% CO_2_ incubator, the macrophages were collected and counted. 1x 10^6^ macrophages were administrated into B6 anti-GBM challenged mice through tail vein injection.

### RNA isolation and mRNA microarray

To compare the gene expression profile between 129x1/svJ macrophages and B6 macrophages, we isolated total RNA from both B6 and 129x1/svJ macrophages using RNeasy Micro Kit, and these RNA samples were further purified over RNeasy Qiagen columns (Qiagen Inc., Germantown, MD). Microarray analysis and quality control steps were performed using validated protocols prescribed by the UT Southwestern Microarray Core (http://microarray.swmed.edu). Simply, purified biotin-labeled cRNA was generated and hybridized onto Affymetrix arrays, HG-U133A, using the Affymetrix protocol according to the manufacturer’s instructions. Each chip was scanned and subjected to Affymetrix algorithm analysis, normalized to the mean intensity of all values and subjected to further analysis.

R version 4.0.3 with the readxl, stats, gplots, readr and ggplot2 packages were used for RNA microarray data analysis. The genes were classified as significantly elevated if they had a p value <0.05 and a fold-change > 5. The Mann-Whitney Wilcoxon test and the Student’s t-test were performed using the respective R packages. Heatmaps were generated for the gene samples that cluster genes with similar expression patterns together. Two heatmaps were generated for this analysis; one heatmap focuses on genes with a mean value of 129x1/svJ BMDMs sample ≥ 42 using the log_10_ transform of gene levels. The second focuses on genes that were significantly elevated if they had a p value <0.05 and a fold-change >5, using a *t*-test. The data was imported to R for cluster analysis and heatmap generation. For clustering, genes were clustered based on correlation distance and average linkage.

Gene Ontology (GO) enrichment analysis was also performed on genes that were significantly elevated if they had a p-value <0.05 and a fold-change >5, using t-test. The genes were analyzed for enrichment in three GO ontologies: biological process, cellular component, and molecular function. Top GO categories with an adjusted enrichment p value of less than 0.01 and fold change more than 1.5 using Fisher’s Exact test are included in the GO Analysis plot. Categories within the same process were ordered by log_10_ transformed p-values. In addition, Integrated Pathway Analysis was performed to identify pathways that were significantly elevated in 129x1/svJ macrophages when compared to B6 macrophages.

### Statistical analysis

Statistical significance was assessed using Prism3 software (GraphPad Software, San Diego, CA, USA). Paired data were analyzed using a paired t-test. Three or more data sets were compared using one-way ANOVA, and the difference between two groups was further compared using Tukey’s multiple comparison test. Data are presented as the mean ± SD. *P* value less than 0.05 was considered statistically significant.

## Results

### Immune cells, not intrinsic renal cells, trigger renal injury in anti-GBM nephritis

**Figure 2:**
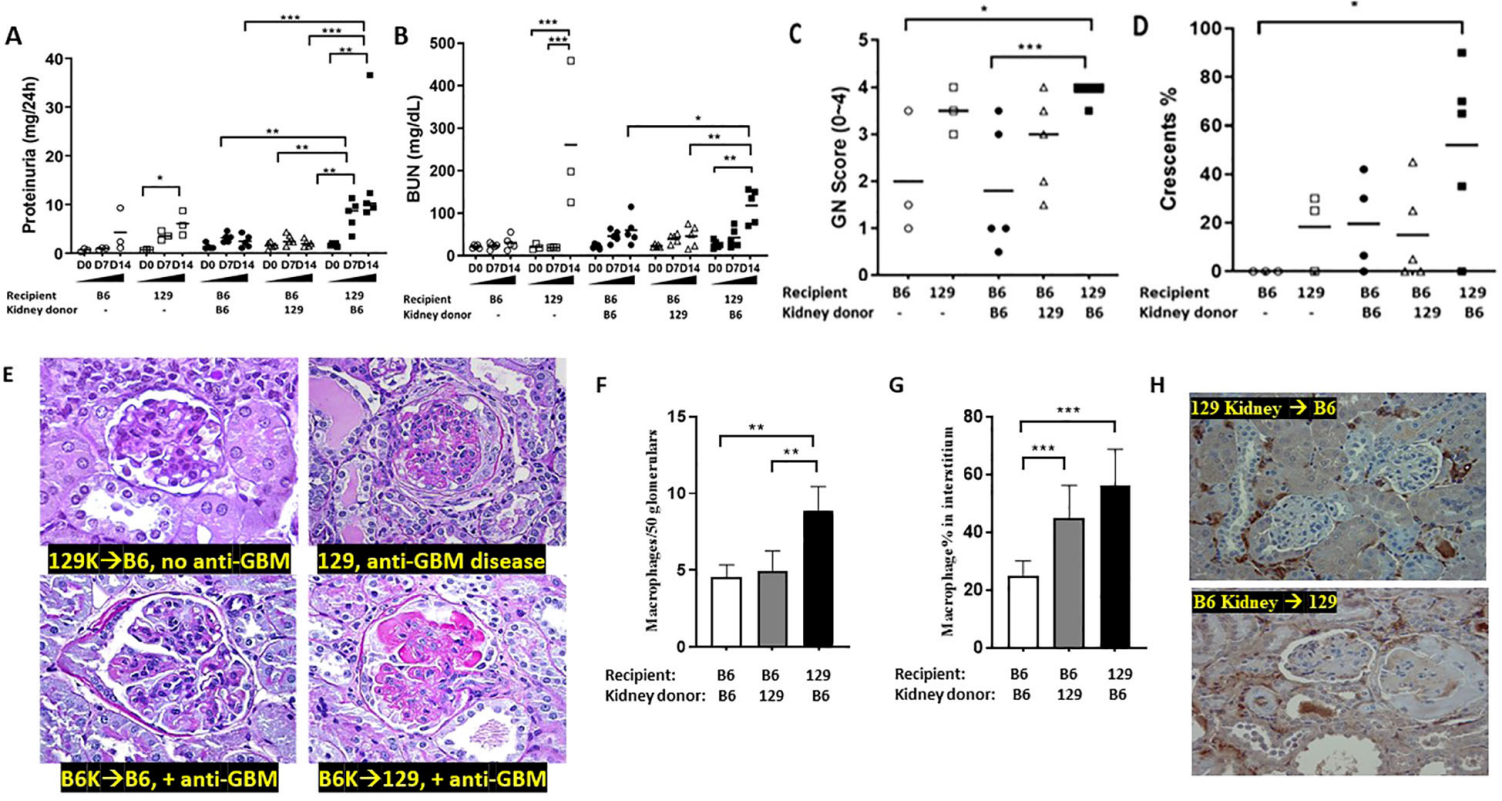
The immune system, not intrinsic renal cells, dictate renal injury in immune-mediated nephritis. Renal transplantation was performed between B6 and 129x1/svJ mice, where the B6 kidney was transplanted into 129x1/svJ recipients or vice versa. 6 weeks after kidney transplantation, transplanted mice with negative proteinuria and a normal BUN were subjected to anti-GBM nephritis. B6 or 129x1/svJ mice without renal transplantation subjected to anti-GBM disease served as controls. Images show: **(A)** 24hr proteinuria levels; **(B)** serum BUN levels; **(C)** GN score; **(D)** the percentage of crescents; **(E)** Representative PAS images from allograft, 129x1/svJ anti-GBM group, B6 recipients transplanted with B6 kidneys and subjected to anti-GBM disease, and 129 recipients transplanted with B6 kidneys, and subjected to anti-GBM disease. **(F)** macrophage infiltration in glomeruli; **(G)** macrophage infiltration in interstitium; **(H)** Representative macrophage IHC staining (n= 3-5 per group, one-way ANOVA test, * p <0.05, ** p < 0.01, *** p < 0.001).

B6 strain of mice are relatively resistant, while the 129x1/svJ (“129”) strain develops severe crescentic nephritis when both are subjected to an anti-GBM serum challenge (10). As B6 and 129 strains have similar MHC (H‐2b), we transplanted kidneys between these two strains (129 kidney to B6 recipient or vice versa). The renal function and pathology changes observed in these anti-GBM challenged kidney recipients are demonstrated in **Figure 2**. Compared to B6 recipients (with 129x1/svJ kidney only), 129 recipients (with B6 kidney only) had significantly higher 24-hr proteinuria and BUN level (n = 5 each group, P <0.01, 2-tailed t-test). B6 recipients (with 129x1/svJ kidney only, n=5) had comparable renal function to B6 recipients with B6 kidneys (n=5) or unmanipulated B6 mice, following induction of anti-GBM nephritis, compared to the significant disease observed in 129 recipients (with B6 kidney only) and unmanipulated 129x1/svJ mice subjected to anti-GBM diseases (**Figures 2A, B** respectively). Consistent with the renal function changes, 129 recipients (with B6 kidney only) exhibited severe renal damage, as evidenced by the increased GN score and percentage of crescent formation (**Figures 2C–E**). Together, these data indicate that the 129 immune system, not the kidney, played a dominant role in triggering renal injury after anti-GBM serum insult. Increased macrophage infiltration was also noted in 129 recipients (with B6 kidney only) in both glomerular and tubulointerstitial areas. The data and representative images are shown in **Figures 2F–H**.

### Transferring 129x1/svJ macrophages, not DCs, enhances immune-mediated nephritis

**Figure 3:**
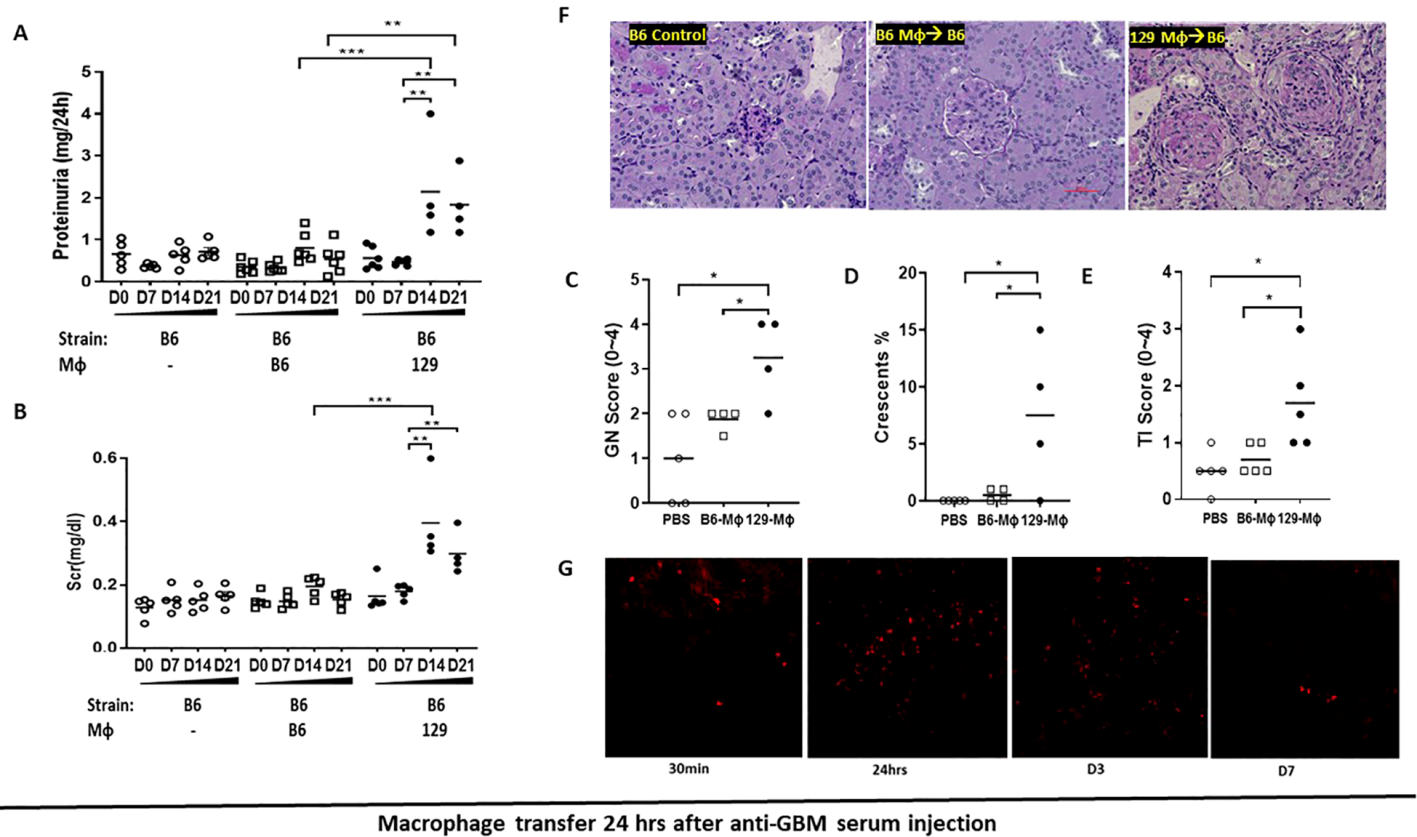
Transferring 129x1/svJ macrophages 24hrs after anti-GBM injection worsens immune-medicated nephritis. Bone marrow-derived macrophages (BMDMs) were generated from B6 or 129x1/svJ bone marrow with MCSF (10 ng/ml) incubation for 5 days. Then 1x106 B6 or 129x1/svJ macrophages were administered to B6 mice 24hrs after anti-GBM serum injection. 129x1/svJ macrophages were labeled with CM-DiI dye to track their renal infiltration. **(A)** 24hr proteinuria levels; **(B)** Scr levels; **(C)** GN score; **(D)** the percentage of crescent formation; **(E)** TI score; **(F)** Representative images from B6 control, B6 mice injected with B6 macrophages; and B6 mice injected with 129 macrophages (from left to right) (N =4-5 each group, one-way ANOVA test, * p <0.05, ** p < 0.01, *** p < 0.001). **(G)** Macrophages homed into the inflamed kidneys following anti-GBM nephritis induction; displayed are representative fluorescent images at 30 min, 24 hr, 3, and 7 days after administration (x200). Renal function and pathology injury were comparable between B6 anti-GBM with or without B6 macrophage transfer. However, compared to the B6 macrophage administration group, B6 mice receiving 129 macrophages showed significant renal dysfunction and pathologic damage, as evidenced by increased 24hr-proteinuria, serum sCr levels, increased GN score, and percentage of crescent formation. (N = 4-5 per group, one-way ANOVA test, and Tukey test, * p <0.05, ** p < 0.01, *** p < 0.001).

The transplantation study indicates that immune cells, not intrinsic renal cells, are critical in driving the development of immune-mediated nephritis. Therefore, we first examined whether macrophages are responsible for initiating renal injury, given their known role in immune mediated nephritis (4–7, 15). We transferred bone-marrow derived 129x1/svJ or B6 macrophages into B6 mice subjected to anti-GBM nephritis, 24 hr before or 24 hours after the anti-GBM serum challenge. In resonance with previous adoptive transfer studies of macrophages in rats (9), our data demonstrated that the mice receiving 129x1/svJ macrophages had higher serum BUN, sCr, and 24-hr proteinuria, with severe renal pathology, compared to mice receiving B6 macrophages (**Figure 3**; **Supplementary Figure 1**, 24 hr after or before anti-GBM serum challenge respectively). As these figures show, renal function and pathology injury were comparable in anti-GBM challenged B6 mice with or without B6 macrophage transfer, irrespective of when the macrophages are transferred (24 hr after or before the anti-GBM serum challenge). However, B6 mice receiving 129 strain macrophages showed significant renal dysfunction and pathologic damage, no matter the 129 macrophages were injected 24 hr after or before the anti-GBM serum challenge (N = 4-5 mice per group, one-way ANOVA test, and Tukey test, * p <0.05, ** p < 0.01, *** p < 0.001).

Next, we explored if 129 strain DCs may also be pathogenic. BM-derived DCs were adoptively transferred into B6 recipients. At baseline, all mice from the B6 DC and 129 DC transfer groups (into B6 recipients) had similar 24-hr proteinuria and serum creatinine levels. 24 hr after anti-GBM serum challenge, either B6-DCs or 129-DCs were IV transferred to these B6 mice. As **Supplementary Figure 2** shows, 24-hr proteinuria and sCr levels were comparable at both D7 and D14 after DC transfer. Thus, in contrast to macrophage transfer, transferring 129x1/svJ bone marrow derived DCs to B6 anti-GBM mice had no impact on renal injury.

### Adoptively transferred macrophages home to inflamed kidneys

Since macrophage transfer elicits renal damage in anti-GBM disease, one critical question is whether these macrophages can traffic to inflamed kidneys and function locally. To explore this, one million fluorescently labeled 129x1/svJ macrophages were injected into B6 via the tail vein one day after anti-GBM serum challenge. Kidneys were collected at 30 min, 24 hr, 3 days, and 7 days after administration. As **Figure 3G** shows, in resonance with previous reports (9), adoptively transferred macrophages home into the inflamed kidney 30 mins after administration, with peak numbers being documented at 24 hr and 3 days post-transfer. However, a trace of fluorescently labeled cells still could still be seen in the kidneys on day 7.

### 129x1/svJ macrophages have an inflammatory phenotype, with enhanced expression of IFN-γ

**Figure 4:**
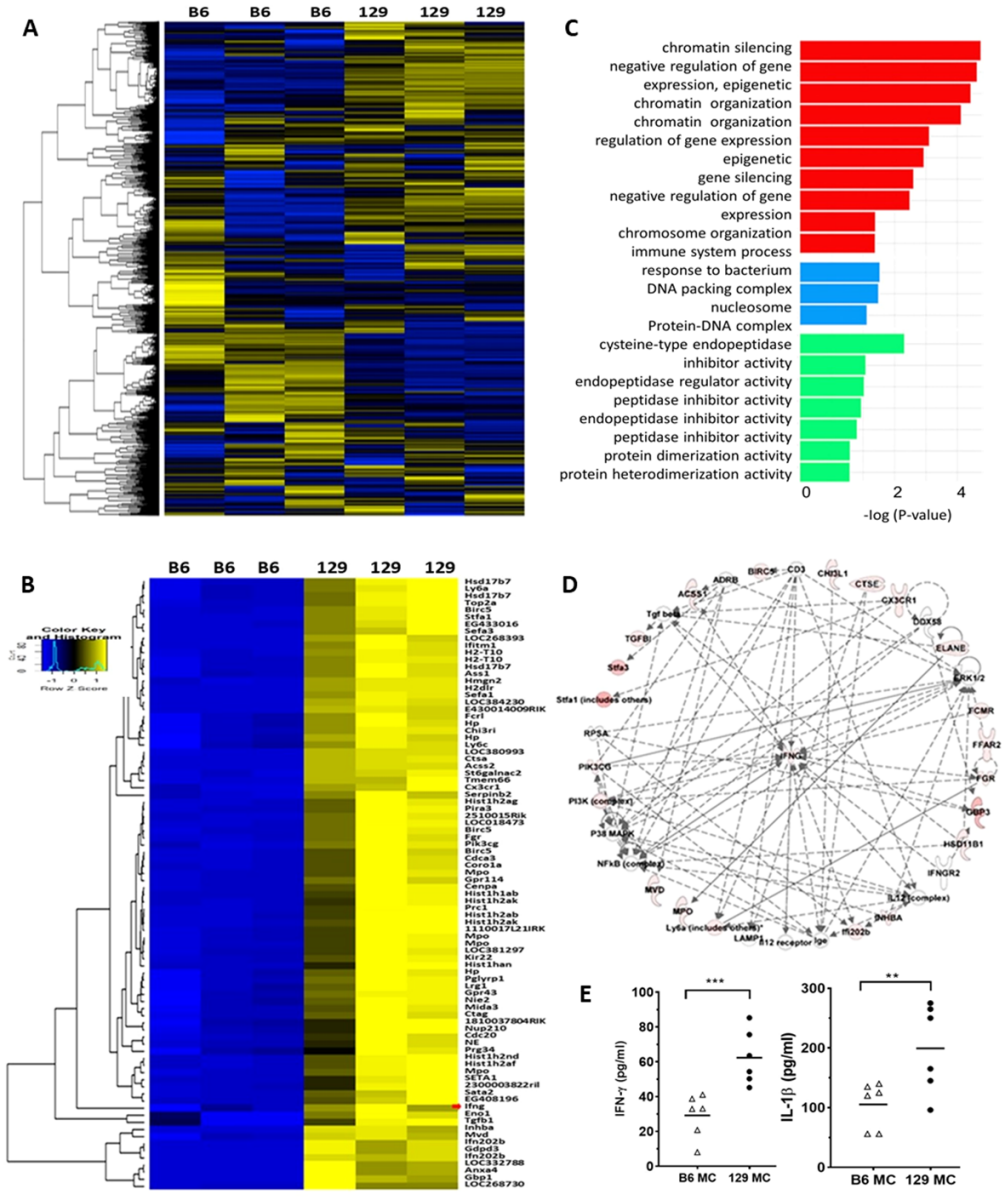
129x1/svJ macrophages exhibit a pro-inflammatory phenotype with up-regulated IFN-γ expression. Total RNA was extracted from B6 and 129x1/svJ bone-marrow-derived macrophages (BMDM), then subjected to gene microarray analysis, and the supernatant was collected for IFN-γ and IL-1β measurement. **(A)** Heatmap representing the expression of 30364 genes, with a mean expression from 129BMDM ≥ 42. Gene intensities were log10 transformed and displayed in colors ranging from blue to yellow. **(B)** Expression heatmap of 86 genes which were elevated in 129 BMDMs, compared to B6 BMDMs (p < 0.05, fold-change ≥5, t-test). The map shows the relative intensities of these genes. The red arrow indicates IFN- γ. For both heatmaps, gene expressions above the mean value are yellow, while those below are blue, with gene expression comparable to the mean value shown in black. Rows are clustered using correlation distance and average linkage. **(C)** Gene Ontology (GO) enrichment analysis for the 86 genes, which were elevated (p< 0.05, fold-change ≥5, t-test) and are mapped in the panther system of gene ontology. These genes were analyzed for enrichment in three GO ontologies: biological process, cellular component, and molecular function. All GO categories with an adjusted enrichment p < 0.01 and fold change > 1.5 are included in the figure, and categories within the same process (color) are ordered by log10 transformed p-value (shown on the x-axis). **(D)** Integrated Pathway Analysis illustrating up-regulated IFN-γ gene expression and its signaling pathway in 129 BMDMs compared to B6 BMDMs. Documented and putative interactions between the displayed molecules are indicated by solid and dashed arrows, respectively. **(E)** The levels of IFN-γ, and IL-1β in the cell culture supernatant of B6 and 129x1/svJ macrophages after LPS stimulation are shown (n= 5 per group, 2-tailed t-test, ** p < 0.01, *** p < 0.001).

Since 129x1/svJ macrophages, but not B6 macrophages, induce severe renal damage, B6 and 129x1/svJ bone marrow derived macrophages were harvested for RNA extraction and subjected to microarray analysis, following TLR stimulation with LPS. From a total of 45,281 genes interrogated in macrophages from three B6 and three 129x1/svJ mice, 30364 genes were noted to have expression levels above noise (mean level in 129 macrophages >= 42). Of these, compared to B6 macrophages, 86 genes were elevated in 129x1/svJ macrophages with p <0.05 and fold-change ≥5, as shown in **Figures 4A, B**. The functions of these differentially expressed genes (DEGs) were also deduced using Gene Ontology functional enrichment analysis. As **Figure 4C** illustrates, the DEGs were related to biological processes such as chromatin silencing, negative regulation of gene expression, epigenetic, and chromatin organization regulation of transcription. For the cellular component and molecular function categories, the most significant enrichment was seen in DNA packing complex and cysteine-type endopeptidase inhibitor activity respectively. Ingenuity Pathway Analysis (IPA) was used to identify putative networks of the interrelated gene, as shown in **Figure 4D**. Increased IFN-γ gene expression and its related signaling pathway was also implicated (**Figures 4B, D**).

Using ELISA, the cell culture supernatant was also collected for IFN-γ and IL-1β measurement. Importantly, 129x1/svJ macrophage secreted more IFN-γ and IL-1β compared to B6 macrophages (**Figure 4E**, n = 5 each group, P < 0.05, 2-tailed t-test).

### IFN- γ mediate the pathogenic effect of macrophage in triggering renal injury

129x1/svJ macrophages showed increased INF-γ and associated gene expression, implicating a potential role of INF-γ in mediating the renal damage seen upon macrophage transfer. In order to test this hypothesis, we upregulated B6 macrophage IFN-γ expression using adenovirus transfer or down-regulated 129x1/svJ macrophage IFN-γ expression using antisense Morpholino oligonucleotides, and then, adoptively transferred these cells into anti-GBM serum-challenged B6 mice.

**Figure 5:**
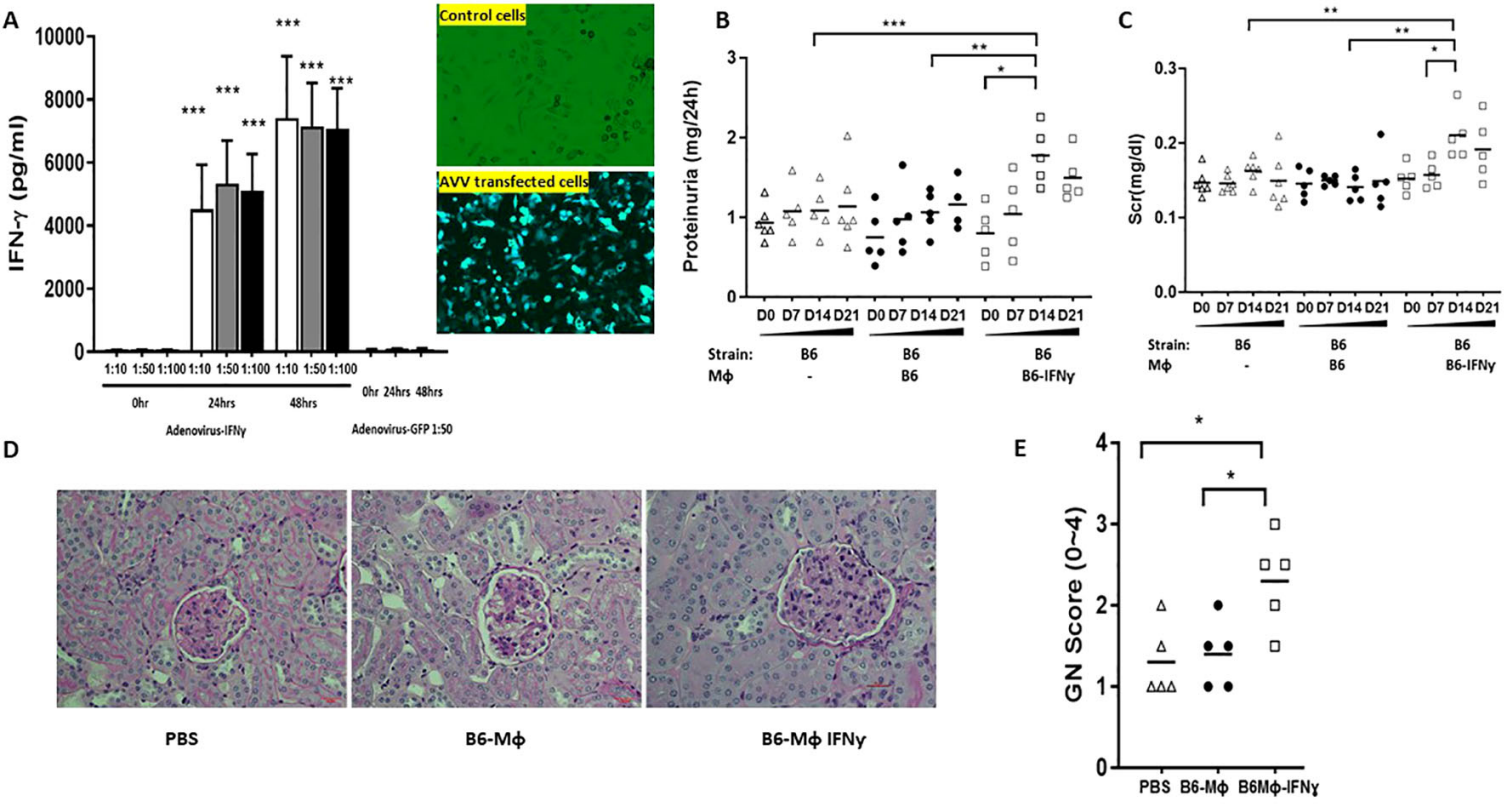
Enhanced IFN-γ expression in B6 macrophages leads to exaggerated immune-mediated nephritis. BMDMs were cultured from B6 bone marrow with MCSF 10ng/ml for 5 days, then transduced with AAV viral particles for 2 days at 50:1 ratio. Non-transduced B6 BMDMs were used as controls. Non-transduced B6 BMDMs or IFN-γ expression enhanced macrophages were administered to B6 mice subjected to anti-GBM disease 24 hrs after anti-GBM serum injection. Displayed are: **(A)** Enhanced IFN-γ production after AAV transfection; **(B)** 24hr proteinuria; **(C)** sCr; **(D)** Representative renal pathology images; **(E)** GN score; Compared to the control and the non-transduced B6 BMDMs groups, the group receiving B6 BMDMs with IFN-γ overexpression exhibited higher 24hr proteinuria **(B)** and sCr levels **(C)** at D14, and increased GN score **(E)** (n= 5-6 per group, two-tailed t-test, * p <0.05, ** p < 0.01, *** p < 0.001).

As **Figure 5A** shows, 24hrs and 48hrs after adenovirus transfection, the supernatant of cultured B6 macrophage exhibited significantly higher IFN-γ. Moreover, IFN-γ specific Morphololino oligonucleotides at 3µM, 6µM, and 10µM all suppressed 129x1/svJ macrophage IFN-γ secretion, as shown in **Figure 1A**.

B6 macrophages with enhanced IFN-γ expression or 129x1/svJ macrophages with suppressed IFN-γ expression were administrated into B6 mice 24hrs after anti-GBM serum challenge, using normal B6 or 129x1/svJ macrophages as controls, respectively. As **Figure 5** shows, compared to the normal B6 macrophage transfer group, mice administered IFN-γ upregulated B6 macrophages had higher 24hr proteinuria (1.776 ± 0.2256 *vs*. 1.067± 0.129 mg/24hrs, respectively, p <0.05), and sCr levels on D14 (0.21 ± 0.07 *vs*. 0.14 ± 0.04 mg/dL, respectively, p <0.01). Consistent with the worse renal function, these mice had increased GN scores (2.3 ± 0.3 *vs*. 1.3 ± 0.3, respectively, p <0.01).

In contrast, mice receiving 129x1/svJ macrophages with subdued IFN-γ expression had lower 24hrs proteinuria (**Figure 1B**, 1.389 ± 0.397 *vs* 2.281 ± 0.595 mg/24hrs on D14; 0.882 ± 0.247 *vs*.1.830 ± 0.858 mg/24hrs on D21, respectively), sCr (**Figure 1C**, 0.164 ± 0.02 *vs* 0.210 ± 0.06 mg/dL on D14; 0.187 ± 0.02 *vs* 0.22 ± 0.03 mg/dL on D21, respectively) on both D14 and D21 (n = 5-6 each group, P < 0.01 two-tailed t-test), compared to normal 129x1/svJ macrophage injection. The GN score of these mice was also lower than that of the 129x1/svJ control macrophage recipients (**Figure 1E**, 1.5 ± 0.3 *vs*.2.4 ± 0.6, respectively, p <0.01). Putting these findings together, our work indicates that enhanced IFN-γ expression in macrophage is pathogenic in nephritis.

## Discussion

Over the last several decades, numerous studies have indicated that renal resident cells are actively involved in the pathophysiology of renal damage in various renal diseases through multiple mechanisms, such as proinflammatory cytokine/chemokine production, cross-talk with immune cells, and epithelial to mesenchymal transition (16–19). In murine crescentic GN, the work of Johnson and his colleagues found that the majority of the cells within crescents were ezrin-positive, but CD4 and CD8 negative, indicating that parietal and visceral glomerular epithelial cells were the main cellular sources of crescents (1). Likewise, using ‘chimeric’ technology, several studies have provided strong evidence supporting the functional contributions of intrinsic renal cells to inflammatory injury in GN via cytokine production and expression of cytokine receptors, MHC-II, and co-stimulatory molecules (2). For example, in a study intending to dissect the contribution of local versus bone marrow (BM)-derived TNF in inflammatory renal injury, Tipping and colleagues reported that intrinsic renal cells were the primary cellular source of TNF contributing to inflammatory injury in crescentic GN (20).

Although the functional contribution of intrinsic renal cells has been well defined, other studies have highlighted the vital role of the immune system in determining renal injury. For instance, glomerular IL-1β production is infiltrating macrophage dependent (21), while IFN-γ from both BM-derived cells and intrinsic renal cells were required to fully develop crescentic GN (22).

Our previous studies demonstrated that the 129x1/svJ strain of mice is susceptible to anti-GBM nephritis, while the B6 strain is relatively resistant (10, 11). The B6 strain shares the same MCH as the 129x1/svJ strain (H‐2b), which allows us to transplant kidneys between these two strains without triggering rejection. The induction of anti-GBM nephritis in these reciprocal kidney recipients allowed us to differentiate the role of the immune system from that of intrinsic renal cells in initialing renal injury. As our data show, the immune system, not the renal resident cells, initiates and drives renal damage and plays a dominant role in triggering the pathogenesis of immune-mediated renal diseases.

Macrophages have long been recognized as a vital mediator of various renal diseases (6, 7, 23, 24). Macrophage infiltration is a common pathological feature of all human chronic renal diseases and rodent nephritis models, and the degree of renal macrophage infiltration is correlated with the severity of diseases and outcome (4, 6, 7). Indeed, several studies have demonstrated that infiltrating macrophages were active and actively contributed to the pathogenesis of renal injury through various mechanisms (6, 24, 25). Consistent with these observations, depleting macrophages through a variety of strategies, including genetic engineering, neutralizing Abs, immunosuppressive compounds, and signaling pathway blockade, have proven to be effective in improving renal function and pathology in several animal renal disease models or patients with renal diseases (23, 24).

For instance, in an experimental rat model of human focal segmental glomerulosclerosis, Wang and colleagues found that adoptively transferring LPS-activated macrophages exacerbated renal injury. However, administering resting macrophages had no significant effect on either renal histology or function (9). Similar to these findings, our data reveal that transfer of 129x1/svJ macrophages, but not B6 macrophages, had a significant impact on renal pathology and function, irrespective of whether the 129x1/svJ macrophages were administered 24 hrs before or after anti-GBM serum injection (**Figure 3**; **Supplementary Figure 1**). Moreover, these 129x1/svJ macrophages could home into inflamed kidneys (**Figure 3G**). In contrast, DCs from both 129x1/svJ and B6 mice, had no significant impact on renal pathology and function (**Supplementary Figure 2**).

The gene expression and singling pathway analysis further demonstrated the pro-inflammatory phenotypes of 129x1/svJ macrophages and revealed gene expression profile differences between 129x1/svJ and B6 macrophages (**Figure 4**). Among these genes, 86 genes were elevated in 129x1/svJ macrophages with a fold change of more than 5 when compared to B6 macrophages, as shown in **Figure 5B**. These genes include IFN-γ, and many IFN-γ regulated genes, such as *Cx3cr1, Birc5, Gbp3, Ifitm1, Klf4, Stat1*, and *TGF-β* (**Figure 4B**). Furthermore, IPA analysis also identified enhanced IFN-γ gene expression and its related signaling pathway in 129x1/svJ macrophages (**Figure 4C**). In line with our gene expression data, a wealth of studies has revealed the role of IFN-γ-regulated genes in regulating macrophage-mediated inflammatory responses. For example, Klf4 has been recognized as a mediator of proinflammatory signaling in macrophages (26–30).

Indeed, we show that B6 macrophages engineered to hyper-express IFN-γ worsen anti-GBM nephritis (**Figure 5**), while down-regulating IFN-γ expression in 129x1/svJ macrophages alleviates disease (**Figure 1**). Consistent with our observations, modulating macrophage activation by IFN-γ has been reported to substantially augment macrophage-mediated renal injury in rat anti-GBM nephritis, independent of any effect on other leukocytes or intrinsic renal cells (31). Likewise, in a mouse model of anti-GBM nephritis, the inhibiting effect of lipoxin on neutrophil infiltration was associated with IFN-γ-induced gene expression (32). Dufour and colleagues provided direct evidence showing how IFN-γ controlled macrophage differentiation and activation in initiating and driving inflammation. They found that C-terminal proteolytic truncation of IFN-γ at 135Glu↓Leu136 could effectively prevent IFN-γ receptor-binding, and this attenuated classical activation of macrophages (33). However, the work of other groups also demonstrated the complicated roles of IFN- γ in deriving and regulating the development of anti-GBM nephritis. A potential protective role of IFN- γ in immune-mediated CresGN has been reported (34, 35). Similarly, Hass and his colleagues reported that mice with IFN- γ receptor deficiency still developed anti-GBM nephritis and argued that IFN- γ was not essential for glomerular crescent formation in mice (36).

In summary, using reciprocal renal transplantation, our work provides unequivocal evidence that the immune system, not intrinsic renal cells, plays a critical role in initiating immune-complex mediated renal injury. Through adoptive cell transfer, we show that macrophages are the dominant immune players accounting for the increased renal disease observed in 129x1/svJ mice. Furthermore, our work demonstrates that IFN-γ is one of the key molecules in controlling macrophage activation and modulating macrophage-mediated renal injury.

## Data Availability

The datasets presented in this study can be found in online repositories. The names of the repository/repositories and accession number(s) can be found in the article/**Supplementary Material**.

## Supplementary material

Supplementary Figure 1 Transferring 129x1/svJ macrophages 24hrs before anti-GBM injection worsens immune-mediated nephritis. Bone marrow-derived macrophages (BMDMs) were generated from B6 or 129x1/svJ mouse bone marrow with MCSF (10 ng/ml) for 5 days. Then 1x106 B6 or 129x1/svJ macrophages were administered into B6 mice 24 hr before anti-GBM serum injection. Shown are: **(A)** 24hr proteinuria; **(B)** BUN levels; **(C)** GN score; **(D)** TI score; **(E)** Representative images from B6 control, B6 mice with B6 macrophage injection, and B6 mice with 129x1/svJ macrophage injection (from top to bottom) (N = 4-5 per group, one-way ANOVA test, * p <0.05, ** p < 0.01, *** p < 0.001). Renal function and pathology were comparable between B6 anti-GBM with or without B6 macrophage transfer. However, B6 mice receiving 129 macrophages showed significant renal dysfunction and pathology, as evidenced by increased 24hr proteinuria, serum Scr levels, increased GN score, and percentage of crescent formation. (N = 4-5 per group, one-way ANOVA test, and Tukey test, * p <0.05, ** p < 0.01, *** p < 0.001).

Supplementary Figure 2 Dendrite cells do not accentuate renal disease progression in anti-GBM nephritis. Bone marrow cells were isolated from both B6 and 129x1/svJ mice and incubated with IL-4 plus GM-CSF (10 ng/ml; R&D Systems) for differentiating DCs. Then, 1x10^6^ B6 or 129x1/svJ DCs were administered into B6 mice 24hr after anti-GBM serum injection. Shown are: **(A)** 24hr proteinuria; **(B)** sCr levels; **(C)** GN score; **(D)** Representative images from B6 control, B6 mice receiving B6 DCs; and B6 mice receiving 129 DCs (from top to bottom) (n=4-5 per group).
