# Supplementary figures and images for: Infiltrating macrophages and interferon gamma rather than renal genotype dictate heightened crescentic glomerulonephritis
Source: Front Immunol. 2024 Dec 19;15:1484525. doi: 10.3389/fimmu.2024.1484525 (PMC11693704; doi:10.3389/fimmu.2024.1484525)

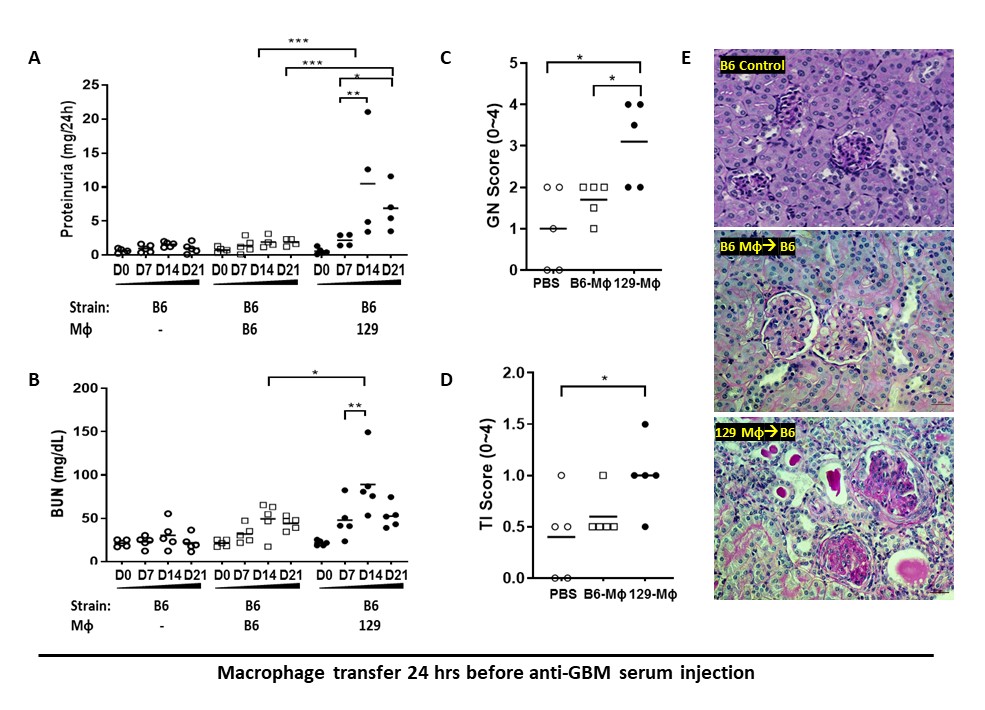

Supplement: Supplementary Figure 1 — Transferring 129x1/svJ macrophages 24hrs before anti-GBM injection worsens immune-mediated nephritis. Bone marrow-derived macrophages (BMDMs) were generated from B6 or 129x1/svJ mouse bone marrow with MCSF (10 ng/ml) for 5 days. Then 1x10^6^ B6 or 129x1/svJ macrophages were administered into B6 mice 24 hr before anti-GBM serum injection. Shown are: (A) 24hr proteinuria; (B) BUN levels; (C) GN score; (D) TI score; (E) Representative images from B6 control, B6 mice with B6 macrophage injection, and B6 mice with 129x1/svJ macrophage injection (from top to bottom) (N = 4-5 per group, one-way ANOVA test, * p <0.05, ** p < 0.01, *** p < 0.001). Renal function and pathology were comparable between B6 anti-GBM with or without B6 macrophage transfer. However, B6 mice receiving 129 macrophages showed significant renal dysfunction and pathology, as evidenced by increased 24hr proteinuria, serum Scr levels, increased GN score, and percentage of crescent formation. (N = 4-5 per group, one-way ANOVA test, and Tukey test, * p <0.05, ** p < 0.01, *** p < 0.001). [file Image1.jpeg]

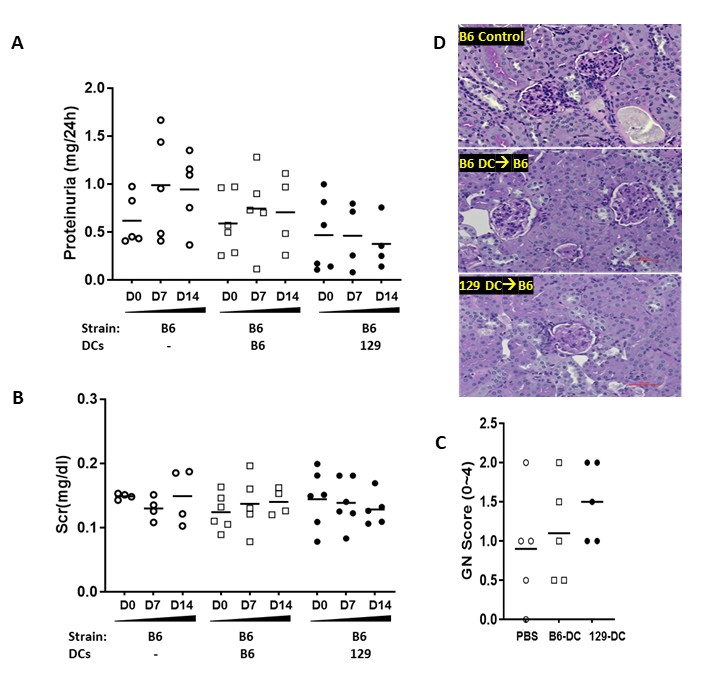

Supplement: Supplementary Figure 2 — Dendrite cells do not accentuate renal disease progression in anti-GBM nephritis. Bone marrow cells were isolated from both B6 and 129x1/svJ mice and incubated with IL-4 plus GM-CSF (10 ng/ml; R&D Systems) for differentiating DCs. Then, 1x10^6^ B6 or 129x1/svJ DCs were administered into B6 mice 24hr after anti-GBM serum injection. Shown are: (A) 24hr proteinuria; (B) sCr levels; (C) GN score; (D) Representative images from B6 control, B6 mice receiving B6 DCs; and B6 mice receiving 129 DCs (from top to bottom) (n=4-5 per group). [file Image2.jpeg]
